# Pinin acts as a poor prognostic indicator for renal cell carcinoma by reducing apoptosis and promoting cell migration and invasion

**DOI:** 10.1111/jcmm.16495

**Published:** 2021-04-03

**Authors:** Ming Jin, Dan li, Weihong Liu, Ping Wang, Zhenfei Xiang, Kaitai Liu

**Affiliations:** ^1^ Department of Radiation Oncology The Affiliated Lihuili Hospital Ningbo University Ningbo China; ^2^ Department of Cardiology The Second Hospital of Yinzhou Ningbo China; ^3^ School of Medicine Ningbo University Ningbo China

**Keywords:** apoptosis, cell invasion, cell migration, Pinin, renal cell carcinoma, The Cancer Genome Atlas (TCGA)

## Abstract

Pinin (PNN) was originally characterized as a desmosome‐associated molecule. Its function and the mechanism of its regulation in renal cell carcinoma (RCC) are still undefined. Data on PNN expression, clinicopathological features, and prognosis of patients with RCC were obtained from The Cancer Genome Atlas (TCGA) database. Immunohistochemistry revealed high PNN expression in tumour cells. PNN expression showed negative correlation with survival in patients with RCC, acting as an independent prognostic factor in RCC. PNN up‐regulation might be attributed to epigenetic alterations in RCC. Immunofluorescence revealed PNN expression mainly in the nucleus of RCC cells. The transfection of siRNA targeting the PNN gene resulted in enhanced apoptosis, which was detected by flow cytometry, and reduced cell migration and invasion, which were assessed using wound healing and transwell migration assay. Gene set enrichment analysis revealed associations between PNN expression and several signalling pathways involved in cancer progression, as a potential mechanism underlying the carcinogenicity of PNN. The analyses of the Tumor Immune Estimation Resource platform showed significant positive associations between high PNN expression and tumour immune infiltrating cells. PNN may function as an oncogenic factor by reducing apoptosis and promoting cell migration and invasion in RCC.

## 1 INTRODUCTION

Worldwide, the incidence of kidney cancer has been increasing year by year, estimated to account for 4.0% of the total new cancer cases in 2021.^1^ Approximately 90% of renal tumours are renal cell carcinoma (RCC), and approximately 80% of them are clear cell carcinoma.^2^ An analysis of the SEER database indicates that RCC incidence has increased, on average, by 0.6% annually and death rates have decreased, on average, by 0.7% annually during 2006 and 2015 in the United States.^3^ However, because of an insidious onset of symptoms in RCC, a remarkable proportion of patients are often diagnosed with locally advanced and distant tumours, and they have a poor five‐year survival rate of about 10%.^4^, ^5^

Pinin (PNN) was initially identified and characterized as a desmosome‐associated molecule as early as 1992.^6^ The *PNN* gene localizes at 14q21.1 and encodes a 140‐kDa phosphoprotein. It was described as a ‘domain rich in serines’, also known as DRS‐protein.^7^ Desmosome is a type of junctional complex that tether intermediate filaments (IF) to the plasma membrane.^8^ The presence of PNN with the mature desmosome, not being integrated with one, is related to the IF organization and epithelial cell‐cell adhesion.^9^ In this view, according to its function of intercellular adhesion reinforcement, it seems that desmosome‐associated molecules, such as PNN, might be considered as tumour invasion suppressors.^10^ However, recent studies have demonstrated that PNN was overexpressed in several types of cancer, including hepatocellular carcinoma (HCC), colorectal cancer (CRC), ovarian cancer and nasopharyngeal carcinoma, and acted as an oncogenic factor, contributing to disease progression and poor survival.^11^, ^12^, ^13^, ^14^ However, its function and the mechanism of its regulation in renal cell carcinoma (RCC) are still undefined.

In the present study, we aimed to investigate the prognostic role of PNN in RCC and the underlying molecular mechanisms by which PNN regulates tumour progression.

## 2 MATERIALS AND METHODS

### 2.1 Clinical data

The clinicopathological and survival data for 512 patients with RCC were downloaded from The Cancer Genome Atlas (TCGA) database using the UCSC Xena platform for public and private cancer genomics data visualization and interpretation (https://xena.ucsc.edu); our analysis included 512 RCC tissue samples and 72 adjacent normal tissue samples. The study was approved by the Ethics Committee of Ningbo Medical Center Lihuili Hospital. In Table S1, the clinicopathologic features of 512 patients with RCC are shown according to PNN expression.

### 2.2 Immunohistochemistry (IHC) analysis

The expression and subcellular localization of PNN in RCC cells were determined by IHC analysis based on 16 RCC samples and corresponding 16 adjacent normal tissue samples stored at Ningbo Diagnostic Pathology Center. Anti‐PNN antibody (Abcam, Hong Kong, China) was used. The proportion score and the intensity score were semi‐quantitatively measured as previously described.^15^

### 2.3 Cell culture

Human clear cell RCC cell lines, OS‐RC‐2 and Caki‐1, were used in the present study; cells were purchased from American Type Culture Collection (Rockville, MD, USA) and cultured in RPMI (HyClone, USA), containing 10% foetal bovine serum (TransGen, Beijing, China) at 37°C, under 5% CO_2_ and saturated humidity.

### 2.4 SiRNAs and immunofluorescence

SiRNAs (siPNN and negative control siRNA) were synthesized by Shanghai GenePharma Co. Ltd. (Shanghai, China); an additional empty vector control was used to control for general effects of siRNA expression. SiRNA sequences are shown in Table S2. OS‐RC‐2 and Caki‐1 cells were transfected with siRNAs using Lipofectamine^TM^ 2000 (Invitrogen, USA) according to the manufacturer's instructions. Transfection was repeated in triplicate. Caki‐1 cells were then permeabilized with 0.1% Triton X‐100 (Sigma Aldrich, USA) for 20 min. After blocking with normal goat serum for 1 h, cells were incubated with anti‐PNN antibody overnight at 4°C. On the next day, cells were washed with PBS and then incubated with secondary antibody (Abcam, USA) for 1 h. Finally, cells were visualized under a fluorescence confocal microscope (Olympus, Japan).

### 2.5 Quantitative real‐time PCR (qRT‐PCR)

Total RNA from OS‐RC‐2 and Caki‐1 cells was isolated using TRIZOL (Invitrogen, USA) according to the manufacturer's protocol, and the concentration of extracted RNA was maintained at around 1500 ng/μL. Next, total RNA was reverse transcribed into cDNAs using M‐MLV reverse transcriptase, and the cDNA template was amplified by real‐time RT‐PCR using the SYBR Green PCR Master Mix (Roche, US) according to the manufacturer's instruction. The reaction system and PCR reaction conditions were conducted as previously described.^15^ The relative expression level of PNN was calculated by the comparative CT method (2^−ΔΔCT^).

### 2.6 Flow cytometric assay

Flow cytometry was used to evaluate the effect of PNN on the apoptosis of RCC cells, by staining them with annexin V‐FITC and propidium iodide (PI). Cells were washed and collected by centrifuging at 2000 rpm for 5 min; cells were then incubated with 5 μL of annexin V‐FITC and 10 μL PI for 5 min in the dark. The cells were analysed with a flow cytometer.

### 2.7 Wound healing assay and transwell migration assay

After transfection with siRNAs, wound healing and transwell matrigel invasion assay was performed to evaluate RCC cell migration and invasion. The distance migrated by cells from the edge to the centre of the plate was measured by taking photomicrographs with an inverted phase‐contrast microscope at zero time point and after 24 h. Image‐Pro Plus (version 6.0) was used to quantify wound healing rate by measuring the ratio of the remaining wound area to the initial one. Transwell migration assays to investigate cell invasion were performed as previously described.^16^

### 2.8 Western blotting analysis

Cells were washed and lysed in SDS Lysis Buffer (sigma, USA) to extract total protein. And protein concentration was quantified using a BCA protein assay kit (Beyotime, Shanghai, China) according to the manufacturer's instruction. About 40 μg of total protein was mixed with loading buffer and loaded in each lane of SDS‐PAGE for electrophoresis. Next, proteins were transferred onto a PVDF membrane at 120 mA constant current for 1.5 h. The membrane was blocked with 5% skim milk for 2 h and incubated with anti‐PNN primary antibody (Cell Signaling Technology, USA) overnight at 4°C. After that, the membrane was washed thrice with TBST and incubated with secondary antibody (Boster, Wuhan, China) for 2 h. Finally, the membrane was washed thrice with TBST and exposed to enhanced chemiluminescence substrates to visualize the expression of the target protein.

### 2.9 Gene Set Enrichment Analysis (GSEA)

To investigate the potential molecular mechanism by which abnormal expression of PNN regulates the carcinogenesis of RCC, we analysed the pathways related to PNN expression in RCC samples using GSEA (version 4.1.0; http://software.broadinstitute.org/gsea/index.jsp); PNN expression data were obtained from TCGA.^17^ A false discovery rate <0.05 was used to identify significantly enriched pathways.

### 2.10 Correlation analysis between PNN expression and tumour immune infiltrating cells (TIICs)

To investigate the associations between PNN expression and TIICs, we assessed specific gene(s) correlated with TIICs utilizing an online public platform named Tumor Immune Estimation Resource (TIMER) (https://cistrome.shinyapps.io/timer/). The TIMER web database includes different TIICs such as B‐cells, CD4+ T cells, CD8+ T cells, dendritic cells, macrophages and neutrophils.

### 2.11 Statistical analysis

Data are presented as mean ±standard deviation, and the means of the two groups were compared using the Student's t test. The difference in clinicopathologic characteristics between the groups was analysed using the chi‐square test. Overall survival (OS) was defined as the interval between the date of diagnosis and the date of death from any cause. Recurrence‐free survival (RFS) was calculated from the date of surgery until the date of disease recurrence. OS and RFS curves were generated using the Kaplan‐Meier method, and the log‐rank test was used to assess the significance of the differences. Univariate and multivariate analysis were performed using a Cox regression model. The corrplot package was used to assess the correlation between PNN expression and DNA methylation. The statistical test used in this study was two‐sided, and a *P*‐value <0.05 was considered statistically significant. Statistical analyses were conducted using SPSS software (version 20.0, IBM, Chicago, USA) and GraphPad Prism (version 5.0).

## 3 RESULTS

### 3.1 PNN was highly expressed in RCC tissues and associated with poor prognosis of RCC patients

**FIGURE 1:**
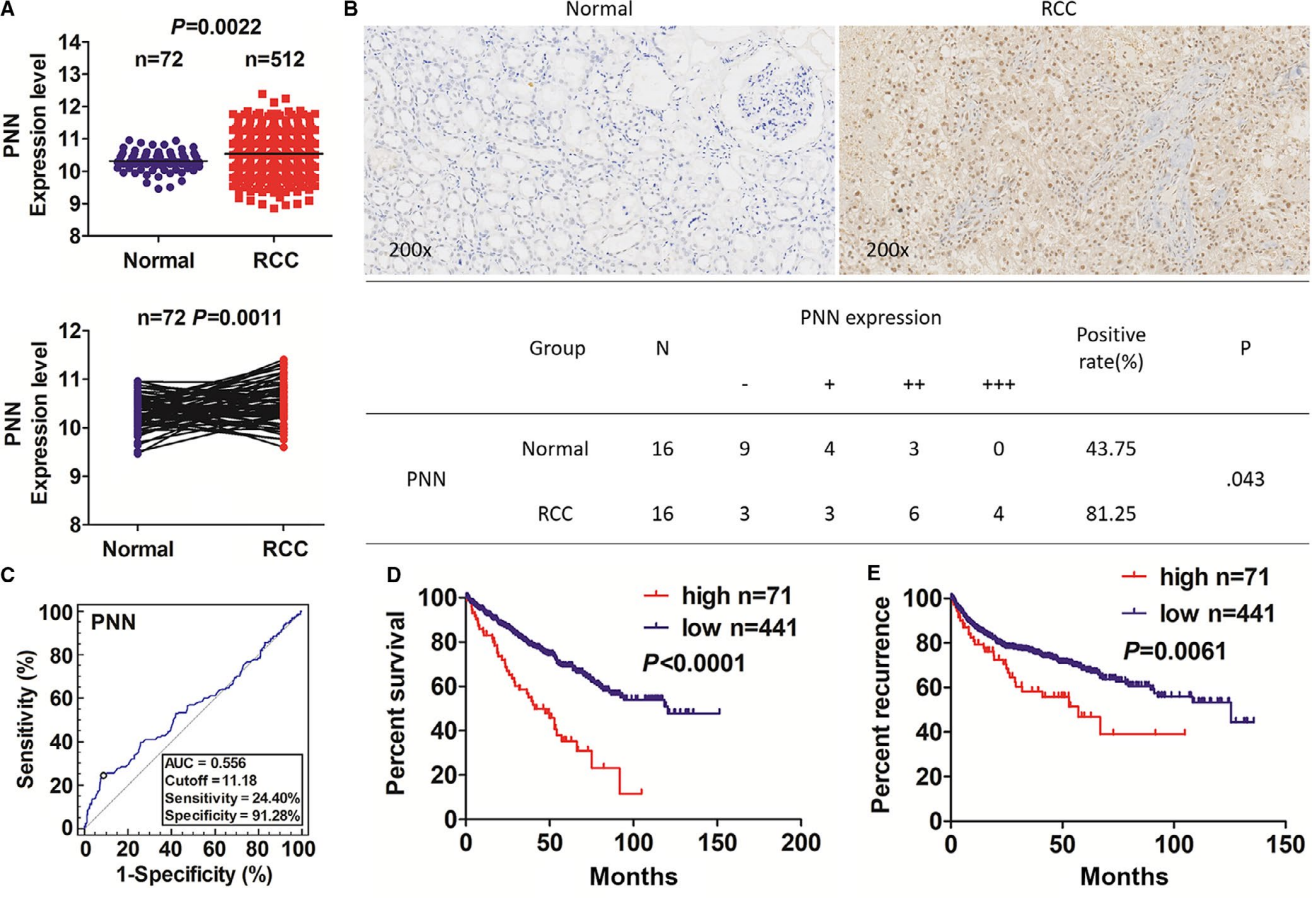
High PNN expression was associated with poor prognosis in patients with renal cell carcinoma (RCC). A, The analyses of The Cancer Genome Atlas (TCGA) data on PNN expression level in the total 512 RCC samples and 72 pair‐matched samples. B, Immunohistochemistry analysis of the expression and subcellular localization of PNN in RCC samples (×200). C, The cut‐off value, sensitivity and specificity of PNN expression in RCC samples (n = 512). D‐E, Kaplan‐Meier survival analyses of the relationship of PNN expression with survival and recurrence time in RCC patients

Our results demonstrated significant up‐regulation of PNN in RCC samples (n = 512) compared with that in adjacent normal tissue samples (n = 72) using TCGA sequencing data. Analyses of the pair‐matched samples consistently showed a marked increase in PNN expression in RCC tissues (n = 72) (Figure 1A). Furthermore, immunohistochemical assay using 16 RCC samples revealed PNN expression mainly in the nucleus; cancer tissues showed significantly higher positive rate in immunohistochemical assay than corresponding adjacent normal tissues (81.25% vs. 43.75%, *P* =.043) (Figure 1B). Moreover, PNN expression level was significantly higher in female patients than that in male patients (*P* =.031); patients with pathological stage III and IV (*P* =.036), stage T3 and T4 (*P* =.046), and metastasis positive (*P* =.023) showed significantly elevated PNN level (Table S1).

Based on the OS time, survival status, and PNN expression level, we acquired the cut‐off value of PNN expression of 11.18 in RCC samples (n = 512) and divided the patients into two groups: PNN high expression (n = 71) and PNN low expression (n = 441). The area under the receiver operating characteristic curve was used to evaluate the sensitivity and specificity of PNN expression in predicting survival outcome (Figure 1C). Kaplan‐Meier analysis indicated that the patients with high PNN expression had significantly worse survival time and higher tumour recurrence rate than those with low PNN expression (Figure 1D‐E). Furthermore, the survival analyses in patients stratified according to different stages of RCC (early‐stage I+II and late‐stage III+IV) showed the same trend (Figure S1). Univariate and multivariate analyses using a Cox regression model showed PNN expression as an independent prognostic factor for survival and recurrence time in patients with RCC (Table S3‐4).

### 3.2 Negative correlation between PNN expression and DNA methylation in RCC

**FIGURE 2:**
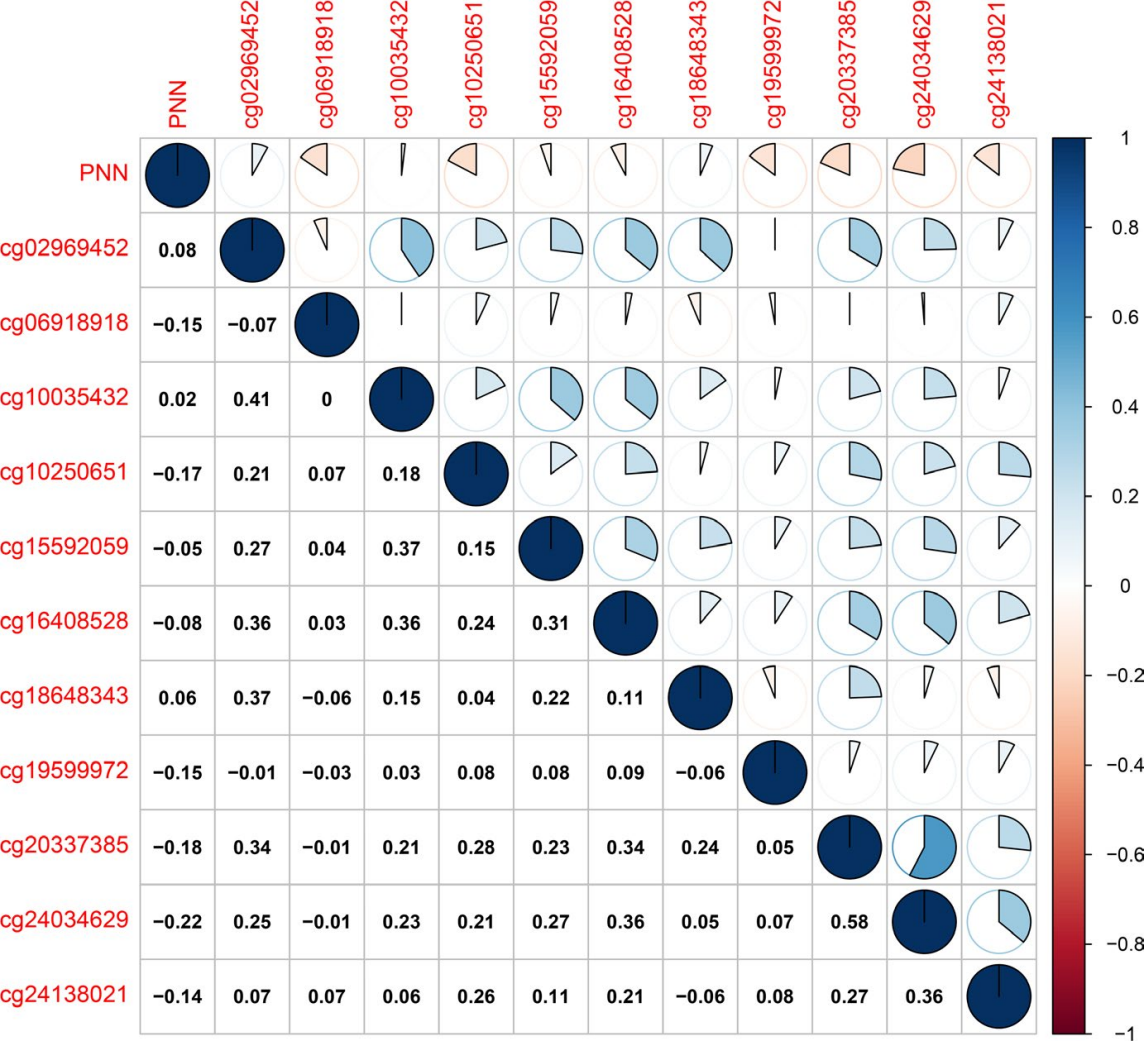
Pearson's correlation between PNN expression and DNA methylation in RCC

To explain PNN up‐regulation in RCC samples, we investigated the epigenetic dysregulation of PNN expression in RCC by analysing DNA methylation data set obtained from TCGA (Illumina HumanMethylation 450K). The results showed that methylation status of 6 out of 11 CG sites assessed in PNN promoter region exhibited negative correlation with PNN expression, indicating that DNA methylation may account for the up‐regulation of PNN in RCC (Figure 2).

### 3.3 PNN down‐regulation resulted in enhanced apoptosis in RCC

**FIGURE 3:**
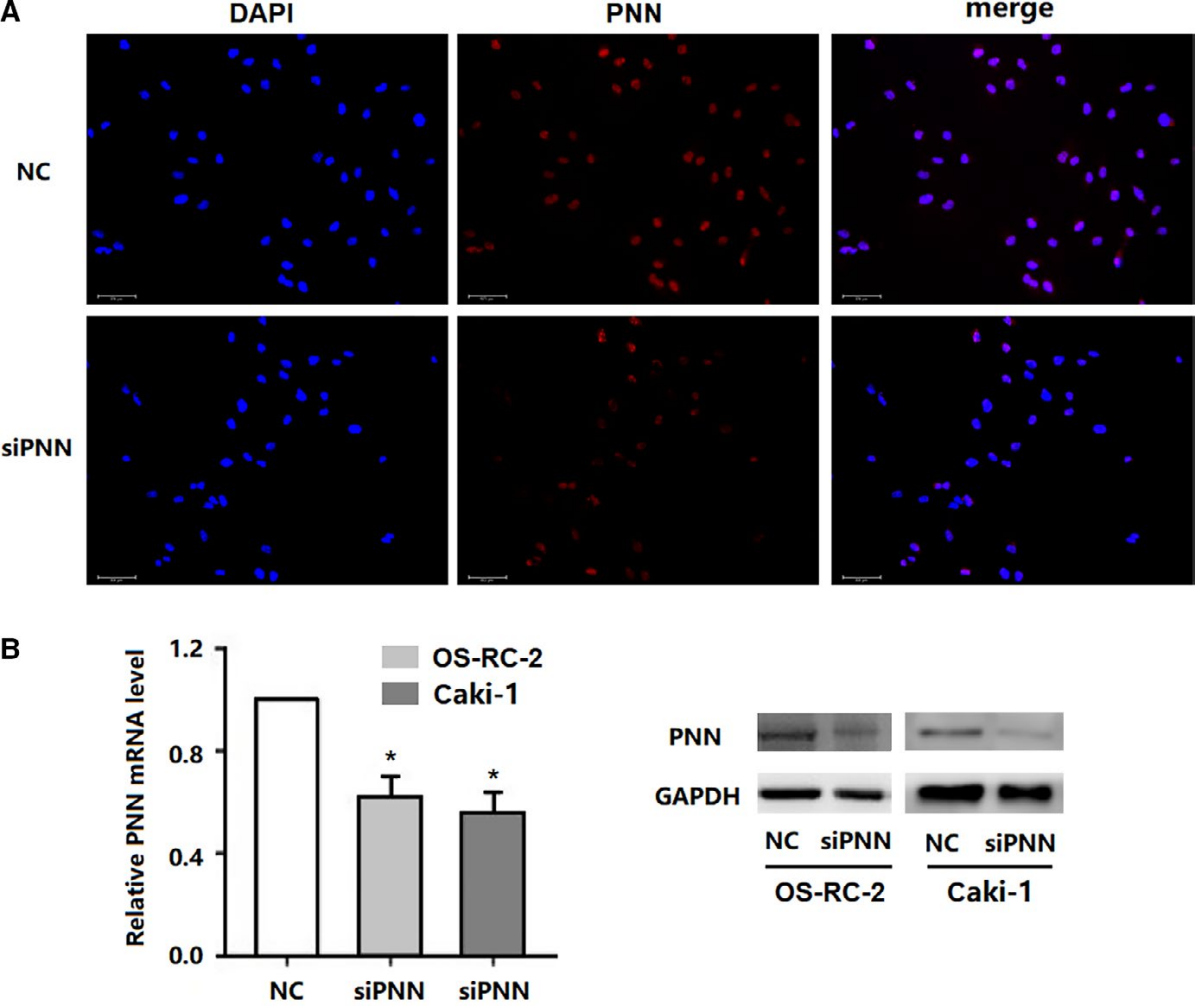
Immunofluorescence and siRNA knockdown of PNN in renal cell carcinoma cell lines. A, Immunofluorescence assay to detect PNN in Caki‐1 cell lines. B, Transfection efficiency of siRNA detected by qRT‐PCR and Western blot analyses. Data are presented as the mean ±standard deviation (SD) calculated from three experiments. *, *P* <.05

**FIGURE 4:**
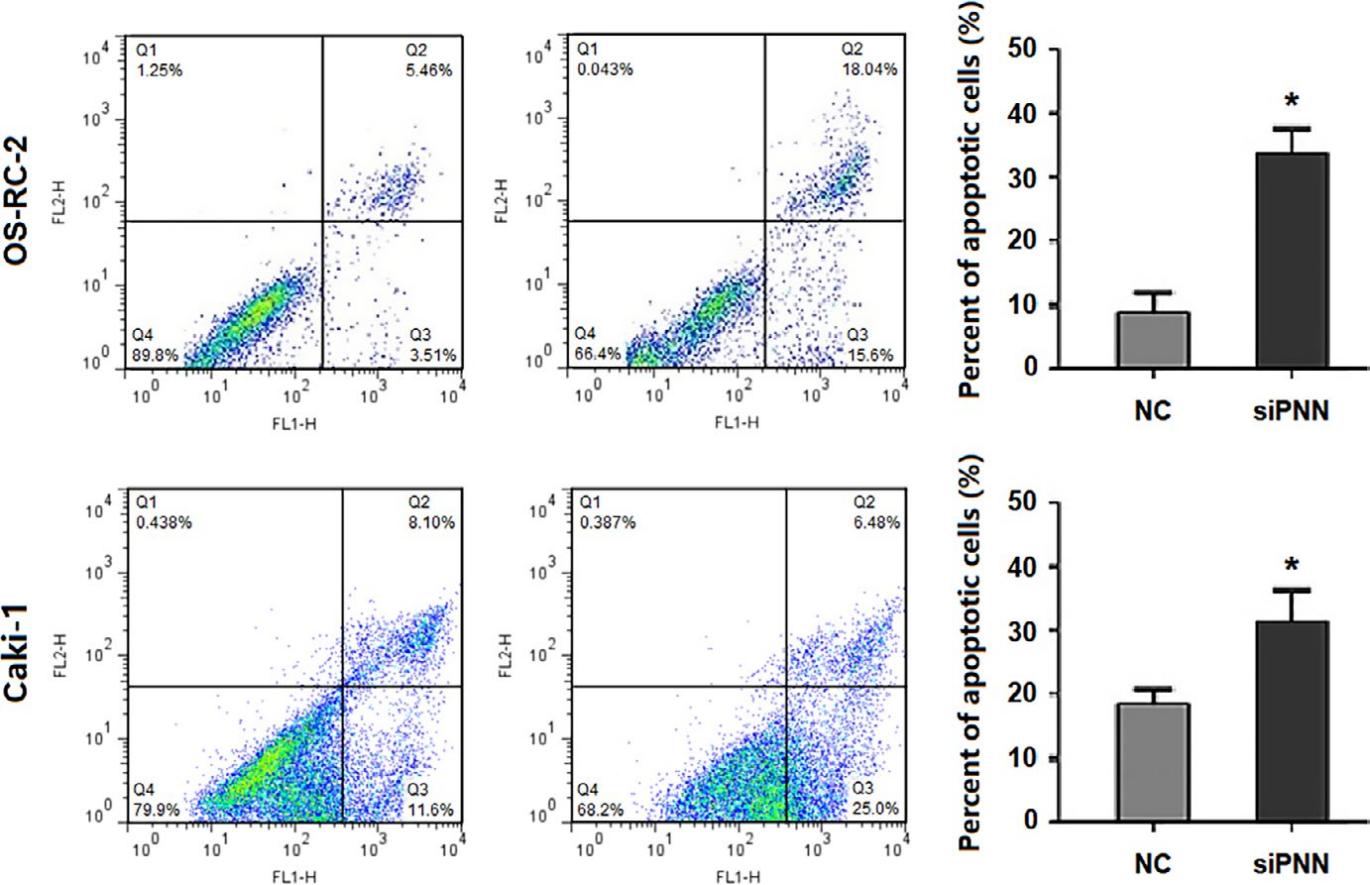
Down‐regulated PNN expression accelerated apoptosis in two renal cell carcinoma cell lines. **, *P* <.01

To investigate the role of PNN in RCC cells, siPNN was transfected into OS‐RC‐2 and Caki‐1 cells. Immunofluorescence assay in Caki‐1 cell lines revealed PNN expression mainly in the nucleus, which obviously decreased in cells transfected with siPNN (siPNN group) compared to that in cells transfected with negative control siRNA (NC group) (Figure 3A). Furthermore, the transfection efficiency of siRNA was assessed by qRT‐PCR and Western blot analyses. The results showed that the expression of PNN was markedly down‐regulated in the siPNN group compared to that in the NC group (Figure 3B). Flow cytometry was used to evaluate apoptosis in siRNA treated RCC cells. Both OS‐RC‐2 and Caki‐1 cells showed markedly higher apoptosis rates in the siPNN group than those in the NC group (*P* <.01), indicating that PNN may reduce apoptosis in RCC cells (Figure 4).

### 3.4 PNN down‐regulation might decrease cell migration and invasion in RCC

**FIGURE 5:**
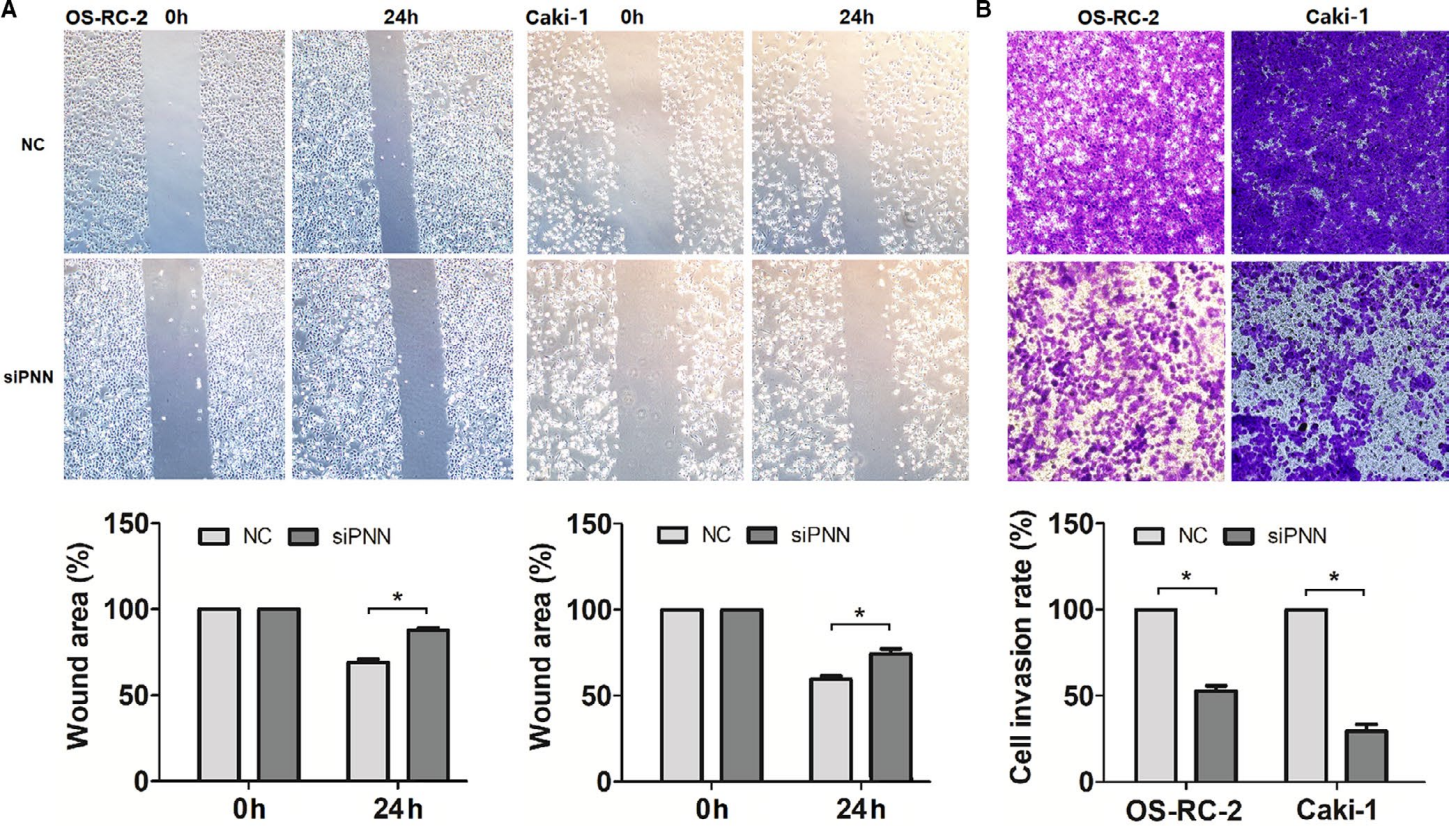
Down‐regulated PNN expression inhibited cell migration and invasion in two renal cell carcinoma cell lines. A, Wound‐healing assay analysing cell migration. B, Transwell migration assay analysing cell invasion. Data are presented as mean ±SD calculated from three experiments. *, *P* <.05; **, *P* <.01

We used wound healing and transwell matrigel invasion assays to investigate the role of PNN in cell migration and invasion in OS‐RC‐2 and Caki‐1 cells. Both OS‐RC‐2 and Caki‐1 cells showed significantly smaller wound area in the NC group than that in the siPNN group (*P* <.05) (Figure 5A). Moreover, the transwell migration assay revealed that the cell invasion rates of both OS‐RC‐2 and Caki‐1 were significantly lower in the siPNN group than those in the NC group (*P* <.05) (Figure 5B). These results suggest that PNN may promote cell migration and invasion in RCC.

### 3.5 Potential regulatory mechanism of PNN in RCC carcinogenesis

**FIGURE 6:**
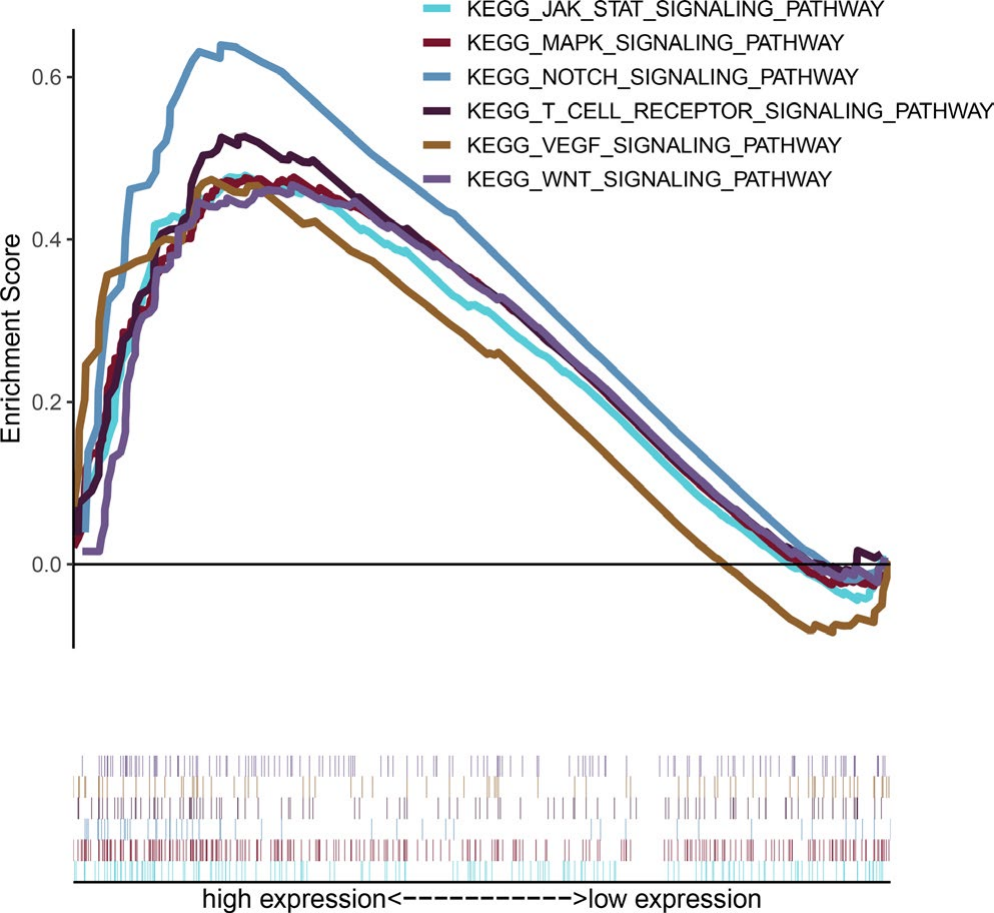
Gene set enrichment analyses (GSEA) revelling the relationship between PNN expression and cancer‐related Kyoto Encyclopedia of Genes and Genomes (KEGG) pathways

We performed GSEA for interpreting high PNN expression with statistically significant prognostic value to evaluate the underlying molecular mechanism of carcinogenicity of PNN in RCC. The result indicated that high PNN expression was significantly related to several signalling pathways, which were believed to be involved in cancer progression, including JAK/STAT, MAPK, Notch, T cell receptor, VEGF and WNT signalling pathways (Figure 6).

### 3.6 Correlations between PNN expression and TIICs in RCC

**FIGURE 7:**
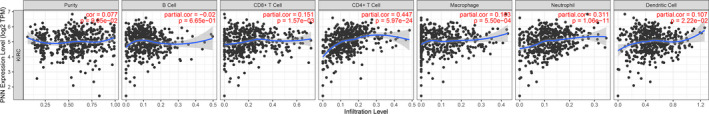
The analyses of the TIMER web database revealing the relationship of PNN expression with tumour purity and TIICs, including B‐cells, CD4+ T cells, CD8+ T cells, neutrophils, macrophages and dendritic cells, in RCC

Analysis of the TIMER database revealed that PNN expression was not significantly correlated with tumour purity and B‐cells. Further analyses demonstrated that PNN expression had markedly positive associations with CD4+ T cells, CD8+ T cells, dendritic cells, macrophages and neutrophils (Figure 7). These results suggest that PNN may promote tumour progression by regulating tumour immune microenvironment in RCC.

## 4 DISCUSSION

PNN is a dual‐location protein classified into desmosome‐form PNN and nucleus‐form PNN.^18^ The desmosome‐form PNN is involved in anchoring intermediate‐sized filaments to the desmosomal plaque, and it affects cell‐cell adhesion.^7^ Joo et al reported that decreased PNN expression could inhibit E‐cadherin and Dsg2, leading to the loss of epithelial cell adhesion and inhibiting human corneal epithelial cell migration.^19^ Further investigation showed that the changes in both PNN location and expression level could influence the epithelial junction and the migration of corneal epithelial cells.^20^ On the other hand, nucleus‐form PNN was characterized as a structural protein involved in pre‐mRNA processing and was shown to play an essential role in alternative RNA splicing.^21^, ^22^ Based on the specific function of PNN in cell‐cell adhesion and alternative RNA splicing, PNN was expected to have a regulatory effect on cancer progression. Shi et al reported that restoration of PNN expression in transformed cells not only positively influenced cellular adhesive properties but also reversed the transformed phenotype to an epithelial‐like morphology.^23^ Further investigation of the potential role of PNN as a tumour suppressor showed low PNN expression in cancer‐derived tissues and cancer cell lines, including epithelial‐derived RCC cell lines, B‐cell lymphoma cell line, and melanoma cell line.^23^ Additionally, the expression and subcellular localization of PNN were found to be altered in various primary tumour tissues. The abnormal expression of PNN in several cancers could be explained by aberrant methylation of CpG islands in PNN promoter region. Moreover, PNN overexpression could inhibit the growth of J82 and HEK‐293 cells. In contrast, recent studies have shown that PNN was overexpressed in tumours and was associated with tumour progression and poor prognosis.^11^, ^12^, ^13^, ^14^ Wei et al reported that up‐regulated expression of PNN was remarkably related to cancer cell proliferation and metastasis in colorectal cancer.^12^ Zhang et al found that down‐regulation of PNN led to inhibition of cell adhesion and clone formation in ovarian cancer.^13^ Tang et al reported that PNN was involved in the AATBC/miR‐1237‐3p/PNN axis and acted as an oncogenic factor in nasopharyngeal carcinoma, contributing to tumour metastasis.^14^ Furthermore, some studies showed that PNN might cause resistance to chemotherapy, such as paclitaxel in ovarian cancer and fluoropyrimidine in colon cancer.^24^ Given the inconsistency in PNN expression and function in cancers reported by previous studies, we aimed to investigate the subcellular localization and expression of PNN in RCC samples in the present study. We showed markedly increased PNN expression in RCC samples compared with that in adjacent normal tissues. We observed PNN expression mainly in the nucleus in RCC tumour cells. Our results indicate that PNN acts as an independent prognostic factor for survival and recurrence time in RCC patients by reducing apoptosis and promoting cell migration and invasion in RCC. In accordance with the findings of Shi et al, our results indicated that DNA methylation might be one of the mechanisms underlying the aberrant expression of PNN in RCC.

Up to now, the molecular mechanism by which PNN acts as an oncogenic factor has been poorly understood. According to a previous report, the function of PNN was associated with phosphorylation of ERK. Yang et al reported that high PNN expression reduced apoptosis through maintaining ERK phosphorylation and inhibiting PARP cleavage in HCC.^11^ Wei et al reported that the activation of the EGFR/ERK pathway by PNN had a profound effect on tumour cell proliferation, invasion and metastasis in CRC.^12^ Our previous study revealed the correlation between PNN expression and the PI3K/AKT signalling pathway in RCC.^25^ The activation of AKT could rescue the inhibited effect induced by PNN knockdown. Furthermore, our GSEA results suggest that PNN expression may be related to several signalling pathways involved in tumorigenesis, including JAK/STAT signalling pathway, MAPK signalling pathway, Notch signalling pathway, T cell receptor signalling pathway, VEGF signalling pathway and WNT signalling pathway. Several studies showed that the JAK/STAT signalling pathway and MAPK signalling pathway played a crucial role in proliferation, apoptosis, invasion, drug resistance and prognosis of solid tumours.^26^, ^27^, ^28^ A recent study revealed that the activation of the JAK/STAT signalling pathway could affect the proliferation and differentiation of immune cells as well as survival outcome in acute lymphoblastic leukaemia.^29^ Another study showed that cancer patients might benefit from the treatment using immune checkpoint inhibitor combined with MAPK inhibitor because of the change in tumour immune microenvironment to overcome drug resistance.^30^ In the present study, it is noteworthy that high PNN expression was markedly related to T cell receptor signalling pathway in RCC. Moreover, the analysis of the TIMER web database showed a significant positive association between high PNN expression and TIICs, providing further evidence for the relationship between PNN expression and immune microenvironment in RCC. Therefore, we suggest the regulation of tumour immune microenvironment as an underlying mechanism by which PNN promotes tumour progression in RCC.

In conclusion, our bioinformatics analysis and experimental results suggest that PNN may act as an oncogenic factor and a poor prognostic indicator in RCC by reducing apoptosis and promoting cell migration and invasion. The present study suggests the associations of PNN expression with several signalling pathways and TIICs as the mechanisms underlying the carcinogenicity of PNN in RCC, which need to be investigated further.

## CONFLICT OF INTEREST

The authors confirm that there are no conflicts of interest.

## AUTHOR CONTRIBUTION

**Kaitai Liu:** Conceptualization (lead); Data curation (equal); Formal analysis (equal); Funding acquisition (supporting); Investigation (equal); Methodology (equal); Project administration (lead); Resources (lead); Software (equal); Supervision (lead); Validation (lead); Visualization (equal); Writing‐original draft (lead); Writing‐review & editing (equal). **Ming Jin:** Conceptualization (equal); Data curation (equal); Formal analysis (lead); Funding acquisition (supporting); Investigation (equal); Methodology (equal); Project administration (equal); Resources (equal); Software (lead); Supervision (equal); Validation (equal); Visualization (lead); Writing‐original draft (equal); Writing‐review & editing (lead). **Dan Li:** Conceptualization (equal); Data curation (equal); Formal analysis (equal); Funding acquisition (supporting); Investigation (supporting); Methodology (equal); Project administration (supporting); Resources (equal); Software (equal); Supervision (equal); Validation (supporting); Visualization (equal); Writing‐original draft (supporting); Writing‐review & editing (supporting). **Weihong Liu:** Conceptualization (supporting); Data curation (supporting); Formal analysis (supporting); Funding acquisition (supporting); Investigation (supporting); Methodology (supporting); Project administration (supporting); Resources (supporting); Software (supporting); Supervision (supporting); Validation (supporting); Visualization (supporting); Writing‐original draft (supporting); Writing‐review & editing (supporting). **Ping Wang:** Conceptualization (supporting); Data curation (supporting); Formal analysis (supporting); Funding acquisition (supporting); Investigation (supporting); Methodology (supporting); Project administration (supporting); Resources (supporting); Software (supporting); Supervision (supporting); Validation (supporting); Visualization (supporting); Writing‐original draft (supporting); Writing‐review & editing (supporting). **Zhenfei Xiang:** Conceptualization (supporting); Data curation (supporting); Formal analysis (equal); Funding acquisition (lead); Investigation (supporting); Methodology (supporting); Project administration (supporting); Resources (supporting); Software (supporting); Supervision (supporting); Validation (supporting); Visualization (supporting); Writing‐original draft (supporting); Writing‐review & editing (equal).

## Supporting information

Figure S1

Table S1‐4

## Data Availability

The data used for bioinformatics analyses in this study are freely available on The Cancer Genome Atlas (TCGA) program website (https://xena.ucsc.edu and https://portal.gdc.cancer.gov/). The interpretation and reporting of these data are the sole responsibility of the authors.
