# Supplementary figures and images for: Pinin acts as a poor prognostic indicator for renal cell carcinoma by reducing apoptosis and promoting cell migration and invasion
Source: J Cell Mol Med. 2021 Apr 3;25(9):4340–8. doi: 10.1111/jcmm.16495 (PMC8093961; doi:10.1111/jcmm.16495)

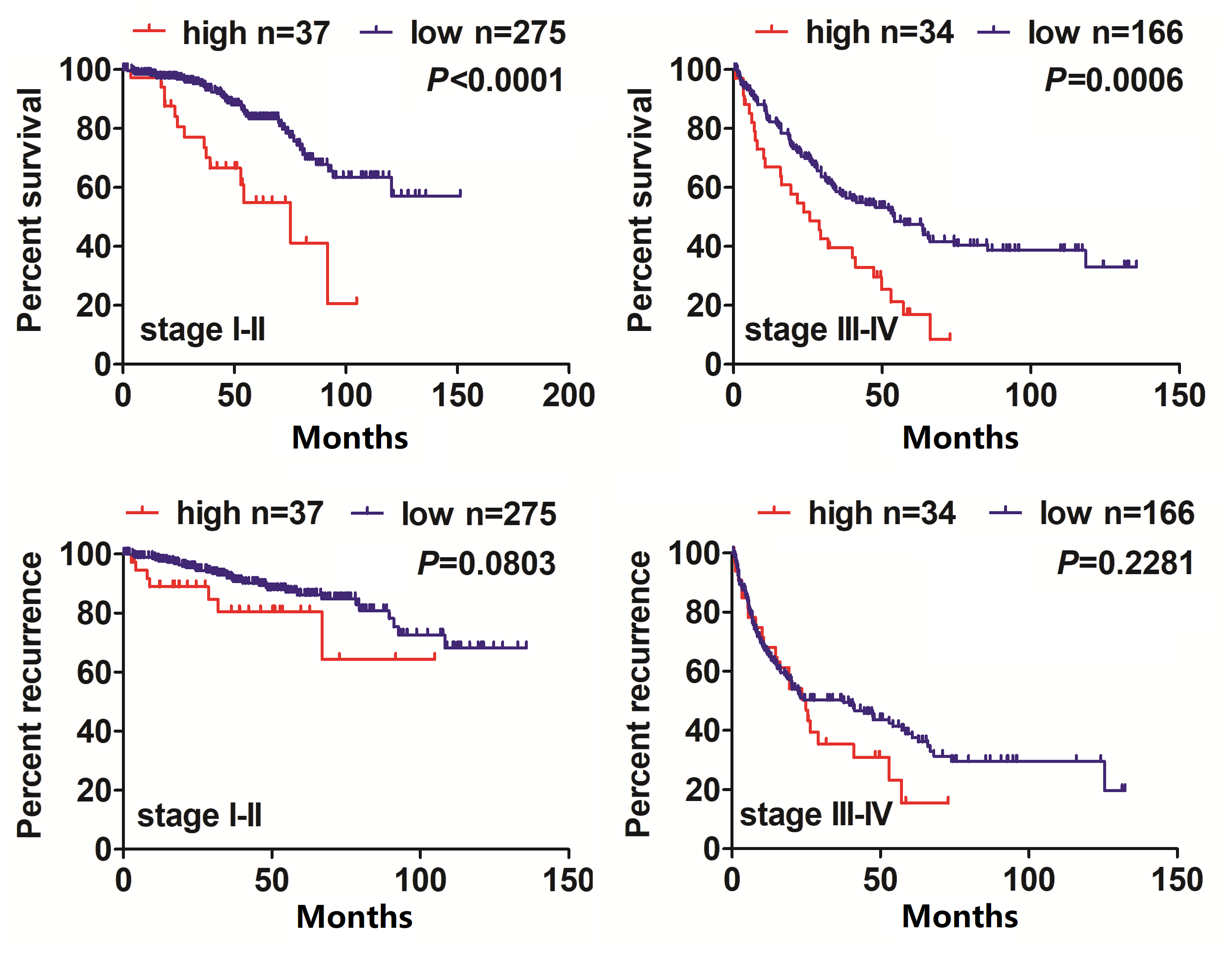

Supplement: Supplementary file 1 — Figure S1 [file JCMM-25-4340-s001.tif]
