# Supplementary material for: Pinin acts as a poor prognostic indicator for renal cell carcinoma by reducing apoptosis and promoting cell migration and invasion
Source: J Cell Mol Med. 2021 Apr 3;25(9):4340–8. doi: 10.1111/jcmm.16495 (PMC8093961; doi:10.1111/jcmm.16495)
Supplement: Supplementary file 2 — Table S1‐4 [file JCMM-25-4340-s002.docx]

**Table S1** The correlation of PNN expression with clinicopathologic

characteristics of RCC patients

| Variables | Cases | PNN | | *P* value |
| --- | --- | --- | --- | --- |
|  |  | High | Low |  |
| Total | 512(%) | 71(%) | 441(%) |  |
| *Age (years)* |  |  |  |  |
| ≥60 | 274(53.5) | 37(7.2) | 237(46.3) | 0.799 |
| <60 | 238(46.5) | 34(6.6) | 204(39.8) |  |
| *Gender* |  |  |  |  |
| Male | 334(65.2) | 38(7.4) | 296(57.8) | 0.031* |
| Female | 178(34.8) | 33(6.4) | 145(28.3) |  |
| *Pathological stage* |  |  |  |  |
| Ⅰ/Ⅱ | 312(60.9) | 35(6.8) | 277(54.1) | 0.036* |
| Ⅲ/Ⅳ | 200(39.1) | 36(7.0) | 164(32.0) |  |
| *T stage* |  |  |  |  |
| T1/T2 | 329(64.3) | 38(7.4) | 291(56.8) | 0.046* |
| T3/T4 | 183(35.7) | 33(6.4) | 150(35.7) |  |
| *N stage* |  |  |  |  |
| Negative | 233(45.5) | 35(6.8) | 198(38.7) | 0.522 |
| Positive | 279(54.5) | 36(7.0) | 243(47.5) |  |
| *M stage* |  |  |  |  |
| Negative | 413(80.7) | 50(9.8) | 363(70.9) | 0.023* |
| Positive | 99(19.3) | 21(4.1) | 78(15.2) |  |

*, *P*<0.05

**Table S2** Sequence of siPNN and NC

| **siRNA** | **Sequence** |
| --- | --- |
| siPNN | sence: 5ʹ-GCA CAC GUA GAG ACC UUA UTT-3ʹ |
|  | antisence: 5-AUA AGG UCU CUA CGU GUG CTT-3ʹ |
| NC | sence: 5ʹ-UUC UCC GAA CGU GUC ACG UTT-3ʹ |
|  | antisence: 5ʹ-ACG UGA CAC GUU CGG AGA ATT-3ʹ |

NC: negative control siRNA

**Table S3** Cox regression analysis of PNN expression as survival predictor

| Variables | Univariate Cox regression analysis | |  | Multivariate Cox regression analysis | |
| --- | --- | --- | --- | --- | --- |
|  | RR (95% CI) | *P* value |  | RR (95% CI) | *P* value |
| *Age (years)* |  |  |  |  |  |
| <60 vs. ≥60 | 1.783 (1.295 to 2.455) | <0.001* |  | 1.633 (1.172 to 2.276) | 0.004* |
| *Gender* |  |  |  |  |  |
| Male vs. Female | 0.993 (0.724 to 1.361) | 0.964 |  | 1.109 (0.794 to 1.548) | 0.544 |
| *Pathological stage* |  |  |  |  |  |
| Ⅲ/Ⅳ vs.Ⅰ/Ⅱ | 3.704 (2.690 to 5.099) | <0.001* |  | 3.362 (1.661 to 6.804) | 0.001* |
| *T stage* |  |  |  |  |  |
| T3+T4 vs. T1+T2 | 3.078 (2.263 to 4.187) | <0.001* |  | 0.748 (0.395 to 1.418) | 0.374 |
| *N staging* |  |  |  |  |  |
| Positive vs. Negative | 0.909 (0.672 to 1.231) | 0.538 |  | 0.853 (0.624 to 1.167) | 0.320 |
| *M stage* |  |  |  |  |  |
| Positive vs. Negative | 3.769 (2.752 to 5.161) | <0.001* |  | 2.176 (1.507 to 3.142) | <0.001* |
| *PNN expression* |  |  |  |  |  |
| High VS. Low | 2.685 (1.880 to 3.834) | <0.001* |  | 2.671 (1.852 to 3.853) | <0.001* |

*, *P*<0.05

**Table S4** Cox regression analysis of PNN expression as recurrence predictor

| Variables | Univariate Cox regression analysis | |  | Multivariate Cox regression analysis | |
| --- | --- | --- | --- | --- | --- |
|  | RR (95% CI) | *P* value |  | RR (95% CI) | *P* value |
| *Age (years)* |  |  |  |  |  |
| <60 vs. ≥60 | 1.328 (0.965 to 1.827) | 0.082 |  | 1.299 (0.936 to 1.803) | 0.118 |
| *Gender* |  |  |  |  |  |
| Male vs. Female | 1.530 (1.076 to 2.175) | 0.018* |  | 1.678 (1.159 to 2.430) | 0.006* |
| *Pathological stage* |  |  |  |  |  |
| Ⅲ/Ⅳ vs.Ⅰ/Ⅱ | 6.596 (4.596 to 9.466) | <0.001* |  | 6.566 (3.375 to 12.776) | <0.001* |
| *T stage* |  |  |  |  |  |
| T3+T4 vs. T1+T2 | 4.448 (3.201 to 6.180) | <0.001* |  | 0.635 (0.360 to 1.119) | 0.116 |
| *N staging* |  |  |  |  |  |
| Positive vs. Negative | 0.878 (0.640 to 1.204) | 0.419 |  | 0.756 (0.546 to 1.047) | 0.092 |
| *M stage* |  |  |  |  |  |
| Positive vs. Negative | 6.401 (4.612 to 8.884) | <0.001* |  | 3.172 (2.185 to 2.604) | <0.001* |
| *PNN expression* |  |  |  |  |  |
| High VS. Low | 1.753 (1.168 to 2.632) | 0.007* |  | 1.744 (1.148 to 2.649) | 0.009* |

*, *P*<0.05
